# Modulating mimicry: Exploring the roles of inhibitory control and social understanding in 5-year-olds' behavioral mimicry

**DOI:** 10.1371/journal.pone.0194102

**Published:** 2018-03-07

**Authors:** Johanna E. van Schaik, Sabine Hunnius

**Affiliations:** 1 Donders Institute for Brain, Cognition, and Behavior, Radboud University Nijmegen, Nijmegen, the Netherlands; 2 Education and Child Studies, Leiden University, Leiden, the Netherlands; TNO, NETHERLANDS

## Abstract

During adult interactions, behavioral mimicry, the implicit copying of an interaction partner’s postures and mannerisms, communicates liking and affiliation. While this social behavior likely develops during early childhood, it is unclear which factors contribute to its emergence. Here, the roles of inhibitory control and social understanding on 5-year-olds’ behavioral mimicry were investigated. Following a social manipulation in which one experimenter shared a sticker with the child and the other experimenter kept two stickers for herself, children watched a video in which these experimenters each told a story. During this story session, children in the experimental group (n = 28) observed the experimenters perform face and hand rubbing behaviors whereas the control group (n = 23) did not see these behaviors. Children’s inhibitory control was assessed using the day-night task and their social understanding was measured through a parental questionnaire. Surprisingly, group-level analyses revealed that the experimental group performed the behaviors significantly less than the control group (i.e. a negative mimicry effect) for both the sticker-sharer and sticker-keeper. Yet, the hypothesized effects of inhibitory control and social understanding were found. Inhibitory control predicted children’s selective mimicry of the sticker-keeper versus sticker-sharer and children’s overall mimicry was correlated with social understanding. These results provide the first indications to suggest that factors of social and cognitive development dynamically influence the emergence and specificity of behavioral mimicry during early childhood.

## Introduction

The characteristics of social interactions are continuously developing during early childhood. As children’s cognitive skills improve and social experiences expand, children become increasingly able to regulate, plan, and coordinate with interaction partners [1–3]. Importantly, such interaction behaviors foster beneficial social consequences, such as peer acceptance, liking, and sharing [4–6]. However, whereas such studies have primarily investigated task-related interaction behaviors (e.g. imitation and coordination tasks), the early development of implicit social behaviors that likely also constitute a part of this social interaction behavior repertoire remains uncertain. Behavioral mimicry is an implicit behavior thought to develop during early childhood, but how social and cognitive development contribute to the emergence of mimicry, and hence mimicry’s role in early social interactions, is unknown.

Behavioral mimicry occurs when interaction partners copy each other’s meaningless behaviors (e.g. rubbing one’s face or bouncing one’s foot up and down) without being explicitly aware of doing so [7]. Adult studies suggest that mimicry is affected by the affiliation goals of the mimicker [8,9]. If participants want to affiliate with their interaction partner, and especially when they have failed to affiliate during a preceding cooperation task, they mimic their interaction partners more [9,10]. The opposite has also been documented: if participants do not like their interaction partner, they tend to “negatively mimic” them by decreasing their own executions of the behaviors performed by their disliked interaction partner [11]. Importantly, the relation between affiliation and mimicry is bidirectional, as being mimicked leads the interaction partner to like the mimicker more, making mimicry an effective tool for implicit affiliation [7,8].

There is some evidence that young children already display behavioral mimicry. In three separate samples, children performed meaningless behaviors, such as cheek scratching and mouth rubbing, significantly more while observing a female video model do so than during a baseline period. While 3-year-olds’ mimicry was unaffected by social manipulations [12,13], 4-6-year-olds primarily mimicked in-group models [12]. This developmental pattern suggests that during early childhood the propensity to mimic becomes more and more a product of the social dynamics of the interaction. It has been put forth that the development of mimicry into the implicit social behavior found in adult interactions is a function of a broadening social understanding and improvements in inhibitory control [12].

Broadly speaking, as children’s social experiences increase during early childhood, so too does their social understanding. This is not only reflected in classic measures of social perspective taking but is also evident from children’s socially sensitive cognition and behavior. A range of false-belief tasks show steady improvement between the ages of two and six [14,15]. Individual differences in performance on such social perspective taking tasks are largely stable, and task performance predicts later peer acceptance [15,16]. Indeed, in adults, mimicry is related to this measure of social understanding, as individuals who score higher on perspective-taking mimic more [7,8]. Increasing awareness of the social dynamics of interactions is further evident in children’s imitative interaction behaviors. Similar to adult mimicry findings, 5- to 6-year-olds imitate in-group members more after being excluded from this group [17]. Correspondingly, particularly 5-year-olds, but to a lesser degree 4-year-olds, display contrasting instead of imitative behaviors in response to out-group members’ behaviors [18]. Children’s explicit understanding of social norms also becomes more influential on their own behaviors with age. While toddlers are already sensitive to receiving an unequal share of resources and tend to equally share resources themselves when all else is equal, it is not until middle childhood that children can overcome preferences for resources and interaction partners to enforce the social norm of sharing on their own sharing behavior [19–21]. In sum, social understanding increasingly influences, and becomes apparent in, children’s cognition and behavior during early childhood, and hence might be related to the increase of socially sensitive interactive behaviors such as mimicry.

Alongside a growing social understanding, young children are increasingly able to regulate their behaviors. A spectrum of inhibitory control tasks show significant improvements between the ages of 2 and 6 years of age [22]. This increasing inhibitory control seems to directly influence children’s interaction skills, as during joint actions inhibitory control is related to children’s accuracy in turn-taking [1]. With respect to behavioral mimicry, these developmental improvements in inhibitory control could be related to the social sensitivity of children’s mimicry that is seen in 4- to 6-year-olds but not 3-year-olds [12]. Hence, it could be the case that, given a level of social understanding that ensures that a child is sensitive to the social interaction context in general, a child’s inhibitory control helps her regulate mimicry such that she is socially selective in who she mimics.

Taken together, behavioral mimicry is a facet of social interactions through which (dis)affiliation is communicated. Although there is evidence to suggest that mimicry develops during early childhood, it is unclear how social-cognitive factors contribute to the emergence of social mimicry. In the current study, we aimed to investigate social mimicry during early childhood and the influences of inhibitory control and social understanding on children’s mimicry. To this end, we designed a partially-live behavioral mimicry paradigm amicable to the use of a social manipulation.

The setup entailed two female experimenters; the first shared one of two stickers with the child (i.e. the sharer) while the second decided to keep both stickers for herself (i.e. the keeper). After this social manipulation, children observed a video in which each experimenter told the child a story. Stories were used as a means of maintaining the children’s visual attention towards the experimenters while minimizing the use of objects that could distract children away from the experimenters. This combination of live interaction and videos was beneficial for several reasons. The social manipulation was live so that it would be experienced as a meaningful interaction for the children and to guarantee that children knew that the experimenters were real individuals, making affiliation possible. The videotaped stories ensured that experimenters did not implicitly act more friendly to some children, controlled the amount of behaviors children were exposed to, and kept the duration of the coded mimicry period constant.

To test mimicry, two groups of participants were used. The experimental group observed the experimenters display behaviors while telling the stories, whereas for the control group the experimenters told stories without displaying these behaviors. This between-participants baseline method ensured that experimental order did not influence behavior prevalence, as the timing of when during the experiment separate baseline and experimental periods occur could influence behavior prevalence due to extraneous factors such as fatigue or initial shyness. Also, arousal during social interactions, particularly in the context of a social manipulation, could be higher than during nonsocial baselines [11], hence this method kept social arousal constant across baseline and experimental measures. Furthermore, a between-participants design also ensured that participants could not experience a contrast of a to-be-mimicked behavior being absent or present during the stories as would be the case in a within-participants design [23].

Social understanding was measured with the short version of the Children’s Social Understanding Scale (CSUS) filled in by the accompanying parent [24]. This scale was selected as it encompasses multiple aspects of social understanding, such as understanding emotions and desires, and concerns everyday examples making it more general and ecologically valid than a standard verbal false-belief task. Inhibitory control was assessed at the end of the experiment using the day-night task [22,25,26]. The day-night task requires participants to inhibit a prepotent response, namely the association between the sun and the concept day and that between the moon and the concept night, in favor of an opposing response, saying “day” when seeing a moon picture and “night” when seeing a sun picture [26].

This design allowed us to test four hypotheses. First, it was hypothesized that, overall, children in the experimental group would display behavioral mimicry, which would be evident in higher behavior occurrences in the experimental group than in the control group. Second, it was expected that children would mimic sharers but not mimic, or negatively mimic, keepers, based on previous evidence that children of this age mimic and can do so selectively [12]. Third, the selectivity of children’s mimicry (i.e. the difference between mimicry of the sharer and mimicry of the keeper) was hypothesized to be a product of inhibitory control. This effect was further expected to be mediated by children’s social understanding, since regulating who you mimic when might require an understanding of who to mimic given the social dynamics. Fourth, social understanding was hypothesized to be positively correlated to children’s overall mimicry, because an increasing experience-based understanding of social interactions would likely lead to increased use of also implicit social behaviors [12].

## Materials and methods

### Participants

Twenty-eight 5-year-olds (60.6 to 61.8 months; mean = 61.29 months; 14 girls) participated in the experimental condition. An additional four children participated but were not included in the final analyses. Two children were excluded prior to data analysis: one because the child’s hands were not visible in the video recording and one because the child had a cast on his arm. Two further participants were excluded during data preparation (see Measures section below). Of the final sample, three children’s day-night scores were scored as missing: one because the child did not want to complete the task, and two due to parental help during the task.

Twenty-three 5-year-olds (60.4 to 61.9 months; mean = 61.03 months; 10 girls) participated in the control condition. An additional six children participated but were not included in the final sample. Five were excluded prior to data analysis; one child had put the sticker on their hand and played with it throughout the session confounding the coding of the hand rub behavior, one had to use the restroom during the story session, one was unable to hear the stories well, one child’s behavior could not be coded due to a corrupt video file, and for one child it could not be ruled out that they were mimicking their parent throughout the session. One additional participant’s data was excluded during data preparation (see Measures section below).

Participants were recruited from a database of volunteer families representative of the middle-sized Western European city the research was conducted in. All parents gave signed informed consent before participation. Children were thanked for their participation with either a children’s book or 10 euros. Additionally, children received a sticker during the social manipulation that they could keep. This line of research was approved by the Ethics Committee of the Faculty of Social Science (ECSW).

### Materials

#### Experimenters

**Fig 1:**
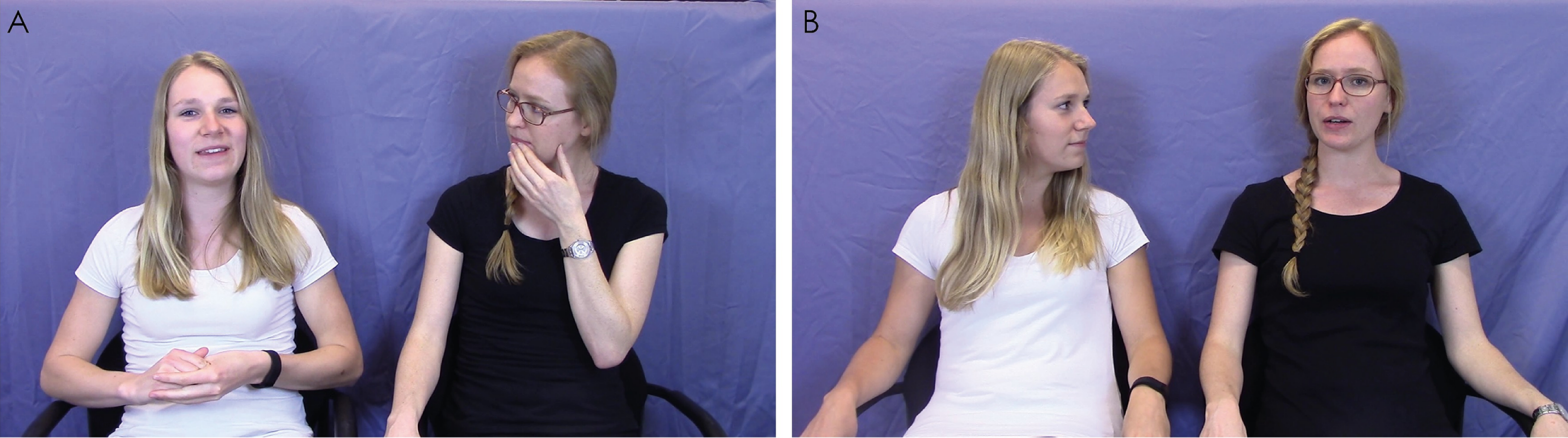
Still frames from story stimuli videos. A: Experimental condition: the experimenter on the left is performing the hand rub behavior while telling her story and the experimenter on the right is performing the face rub. B: Control condition. The researchers pictured in this figure have given written informed consent (as outlined in PLOS consent form) for publication of this figure.

Two female experimenters ran the experiment for all participants (Fig 1). To maximize children’s ability to identify which experimenter was which, one experimenter always wore a white T-shirt, had her hair loose, and sat on the left side of the table (from the child’s point of view), while the other always wore a black T-shirt, had her hair in a braid, wore glasses, and sat on the right.

#### Lab setup

The child sat across from the two experimenters and their parent sat next to them to the side. Each experimenter had a small box with two stickers and a laminated photograph of themselves on their side of the table. Parents were provided with a clipboard with an overview of the procedure and the CSUS. Two unobtrusive synchronized cameras recorded the session and could be monitored in an adjacent room.

#### Day-night task

The task was administered largely following Gerstadt, Hong, and Diamond [26]. First, the experimenter asked the child about when one can see the sun, and if necessary guided the child to the answer “day”, and when the moon is visible (counterbalanced order), and if necessary guided the child towards the answer “night”. Subsequently, the experimenter showed the child one of the cards (e.g. sun) and instructed the child to say “night” when they saw this card and asked them to repeat the word, and then did the same for the other card type. Sixteen trials were presented, of which the first two always included one sun and one moon card. In each trial, the card was placed face-up on the table and the experimenter waited for the child to respond. If the child hesitated, the experimenter asked “What do you say for this card?” If the child responded incorrectly to one or both of the first two trials, the instructions and first two trials were repeated before continuing with the remaining cards. Only after the child had responded correctly to the first two cards did the experimenter continue with the remaining 14. In total, eight cards had a picture of a moon and eight cards had a picture of a sun. The order was pseudo-randomized for every participant. The experimenter did not provide feedback during the session. The other experimenter scored the accuracy of the child’s (first) response per trial from the monitor room. Scores were computed as the sum of trials in which the child responded correctly.

#### CSUS

The short version of the CSUS consists of 18 questions (2 reverse-scored) answered on a scale from”1 Definitely Untrue” to”4 Definitely True” or as “Don’t know”. Questions were translated from English into Dutch by a Native-Dutch speaker and were translated back into English by a bilingual speaker. Differences between the original and back-translated versions were resolved by both translators. Scores were computed by calculating the average over the items, with “Don’t know” items counted as missing, following [24].

#### Stimulus videos

A projector was setup up such that videos could be projected on a white wall directly behind where the experimenters had sat during the live interaction. The stimulus videos, displayed via the projector during the experiment, showed the two experimenters sitting side-by-side in the same manner as during the live interaction (i.e. each experimenter on her respective side with the same attire and appearance; Fig 1). Thus, the video setup closely matched the live interaction from the child’s point of view.

Two types of videos were recorded. In the ‘introduction videos’, one of the two experimenters announced, “Hey, we are sitting here now.” There were two versions of the introduction video, one in which the experimenter on the left spoke and one in which the experimenter on the right spoke. Both experimenters were smiling and looking towards the camera. In the ‘story videos’, the experimenters took turns telling a “Jip and Janneke” story by Annie M. G. Schmidt. The experimenter on the left always told a story about sowing seeds and the experimenter on the right told a story about picking flowers. While one experimenter told the story, the other experimenter looked at her. Both stories were supplemented with four pictures that the storytelling experimenter held up to the camera in order to maintain the children’s attention. These were shown at similar points in the two stories: the first after one-fourth of the story, the second half-way-through, the third after roughly three-quarters of the story had been told, and the final picture near the end of the story.

In the experimental condition, each experimenter carried out one behavior nearly continuously (as is typical of adult mimicry experiments [7,9,10,27]), both while telling the story and while listening to the other tell the story (Fig 1A). In other words, both behaviors were shown throughout the story session, one by one experimenter (e.g. face rubbing) and the other by the other experimenter (e.g. hand rubbing; counterbalanced). The experimenters performed their behaviors in partial overlap, often simultaneously but at times only one was performing a behavior to make the stimuli appear more natural. The face rub consisted of the experimenter freely rubbing her hand over her mouth and chin and scratching her cheek (right and left hands were used interchangeably). On average, face rubs were shown for 2.19 minutes per video. The hand rub consisted of the experimenter rubbing her hands over one another, rubbing her wrists, and interlocking fingers. Hand rubbing was shown an average of 2.25 minutes per video. Behaviors were not carried out by either experimenter while the storytelling experimenter was showing a picture, which were shown for approximately 10 seconds each. In the experimental condition there were four versions of the story videos, as dictated by the four combinations of experimenter order (i.e. first or second) and experimenter’s behavior (i.e. face rub or hand rub); each participant observed one of these versions. An example segment of one of the experimental condition stimulus videos is available in the supporting information (S2 File).

In the control condition, there were two versions of the story video to counterbalance across participants, as no behaviors were carried out (i.e. experimenter 1 tells her story first, experimenter 1 tells her story second; Fig 1B). On average, story videos were 5.5 minutes in duration, with each story taking up about half of that time.

### Procedure

Upon arrival at the lab, the child drew a picture while the experimenters explained the procedure to the parents and showed interest in what the child was drawing by asking questions and complimenting their work. Following this warm-up period, the so-called sticker game (i.e. social manipulation) started. One experimenter (counterbalanced) would open a small box on their side of the table, take out two stickers, and exclaim, “Look! I have two stickers! Wow, these are nice. Look a <picture on sticker, e.g. cat> and a <picture on sticker, e.g. dinosaur>. Wow, these are nice. Do you like them too?” At this point, the child was given the opportunity to answer (all children indeed said they liked the stickers). The experimenter then said, “Well, because I like them so much, I will share them with you. Which one would you like?” Once the child had chosen a sticker, the experimenter put the other sticker down in front of herself and said, “Look, now we both have a sticker!” The other experimenter then repeated this procedure, with the exception that, after asking the child if he/she liked the sticker the experimenter said, “Well, because I like them so much, I will keep both stickers for myself.” The experimenter proceeded to place both stickers in front of her.

Next, the ‘sharer’ gave a little box to the child to put their sticker in so that the child would not have the sticker in her hands during the rest of the experiment. Both experimenters also put their stickers back into their boxes. The sharer then announced that she would have to go to the other room but that she would leave a photograph of herself behind and proceeded to place the photograph facing the child on the table before leaving. After the sharer got up, the ‘keeper’ did the same.

Once the experimenters had left, the parent asked the child, “If you could play another game with one of these two ladies, with who would you want to play? Her or her?” Parents were free to point to the photos of the experimenters and had been instructed to repeat the question until the child made a choice.

Following this, the introductory video was played via the projector. Parents were instructed to move their child’s chair back from the table at this point. This was done so that children would not be leaning on the table nor be able to grasp objects on the table, and to ensure that children’s hands would be visible for offline coding. Once the child was situated, the story video was played. Which experimenter spoke in the introductory video, which experimenter first told her story, and, in the experimental condition, which behavior was carried out by which experimenter, was counterbalanced across participants. Parents were asked to fill in the CSUS while their child watched the story videos.

After the stories, the sharer came back into the room and carried out the day-night task with the child. The session was concluded with the keeper also coming back into the lab. In a funneled debriefing, the keeper asked the child if she had noticed anything while watching the videos and, in the experimental condition, whether she had noticed if the experimenters carried out face rubs or hand rubs. To ensure that the child could identify the experimenters, the keeper asked from whom they had gotten a sticker and from whom they had not (all participants were indeed able to correctly identify the sharer and keeper). The keeper then apologized for not sharing, saying that it was not a very kind thing to do and offered to make up for it by letting the child choose a gift (i.e. the children’s book or money).

### Measures

Video recordings were used to code children’s visual attention, face rubs and hand rubs during the story session, which was performed blind to sharer identity and sharer’s and keeper’s behaviors. Behavior coding requirements followed the modeled behaviors in the stimulus videos (see section above). Hence, face rubs were coded when children rubbed or scratched their faces, with the exclusion of eye rubbing, and hand rubs were coded when children’s hands or fingers were interlocking or moving over one another or their wrists. Behaviors were coded from the first video frame in which there was contact between the hand and target region until the last frame this was the case, if the hand remained lifted for more than one second.

For both groups, the percentage of the story session time that a child carried out a behavior was calculated for both behavior types separately. Behavior percentages as a function of time were selected following the reasoning of Hogeveen and Obhi [28] that percentages measure the absolute presence of a behavior during an interaction. Additionally, a duration-based measure is appropriate since the two behaviors could be carried out continuously (as opposed to, e.g., yawning). Before further measure calculations, behavior percentages were checked for outliers above 3 SDs from the mean per behavior, per group. This led to the exclusion of two participants from the experimental group and one from the control group.

Behavior measure calculations were conducted as follows (see also S1 Table). Within the experimental group, each child’s behavior percentages (i.e. face percent and hand percent) were divided by the control group’s average behavior percent of the corresponding behavior, providing “behavior ratios”. The same was done for the sum of both behaviors to obtain “total mimicry ratios”. Hence, for both the behavior and total mimicry ratios, if a child from the experimental group’s ratio is above 1, the child performed the behavior(s) more than the control group did on average, while if it is below 1, the child performed the behavior(s) less than the control group’s average.

The calculation of behavior ratios allowed for comparisons within the experimental group while correcting for possible general differences in face rubbing and hand rubbing prevalence (as measured in the control group). Thus, this allowed us to collapse across the counterbalancing of face and hand behaviors to test the difference in the mimicry of the sharer versus the keeper, referred to as “social ratios”. Additionally, a measure of “selective mimicry” was calculated per experimental participant by subtracting the keeper ratio from the sharer ratio, providing a difference score indicating how much more (positive values) or less (negative values) the sharer was mimicked than the keeper.

### Comparisons

For all control and main comparisons, assumptions of statistical tests were checked first and non-parametric analyses were used when needed. All reported p-values are two-tailed unless stated otherwise. The measures as well as the inhibitory control and social understanding scores are available as supporting information (S1 File).

#### Control comparisons

To ensure that the two groups of participants did not differ in social-cognitive development, the inhibitory control and CSUS scores were compared. There were no differences between the experimental and control groups in their day-night task score nor their CSUS score (*p*s > .20).

The time children spent watching the videos was near ceiling in both the experimental and control groups, as on average children watched respectively 99.14 and 98.45 percent of the story session. In the experimental group, there was no significant difference between how much of the sharer’s story children watched and how much of the keeper’s story (*p*>.25). This is an important comparison as it indicates that potential differences in the mimicry of the sharer and keeper were unlikely to be a simple effect of having seen one storyteller’s behavior more than the other’s.

Binomial tests were used to test whether the social manipulation affected children’s explicit preference to play with either the sharer or the keeper. In both groups, the observed proportions of children who selected the sharer (experimental group = .59; control group = .41) and keeper (experimental group = .41; control group = .59) did not differ significantly from chance (i.e. 50%; *p*s > .40).

## Results

**Fig 2:**
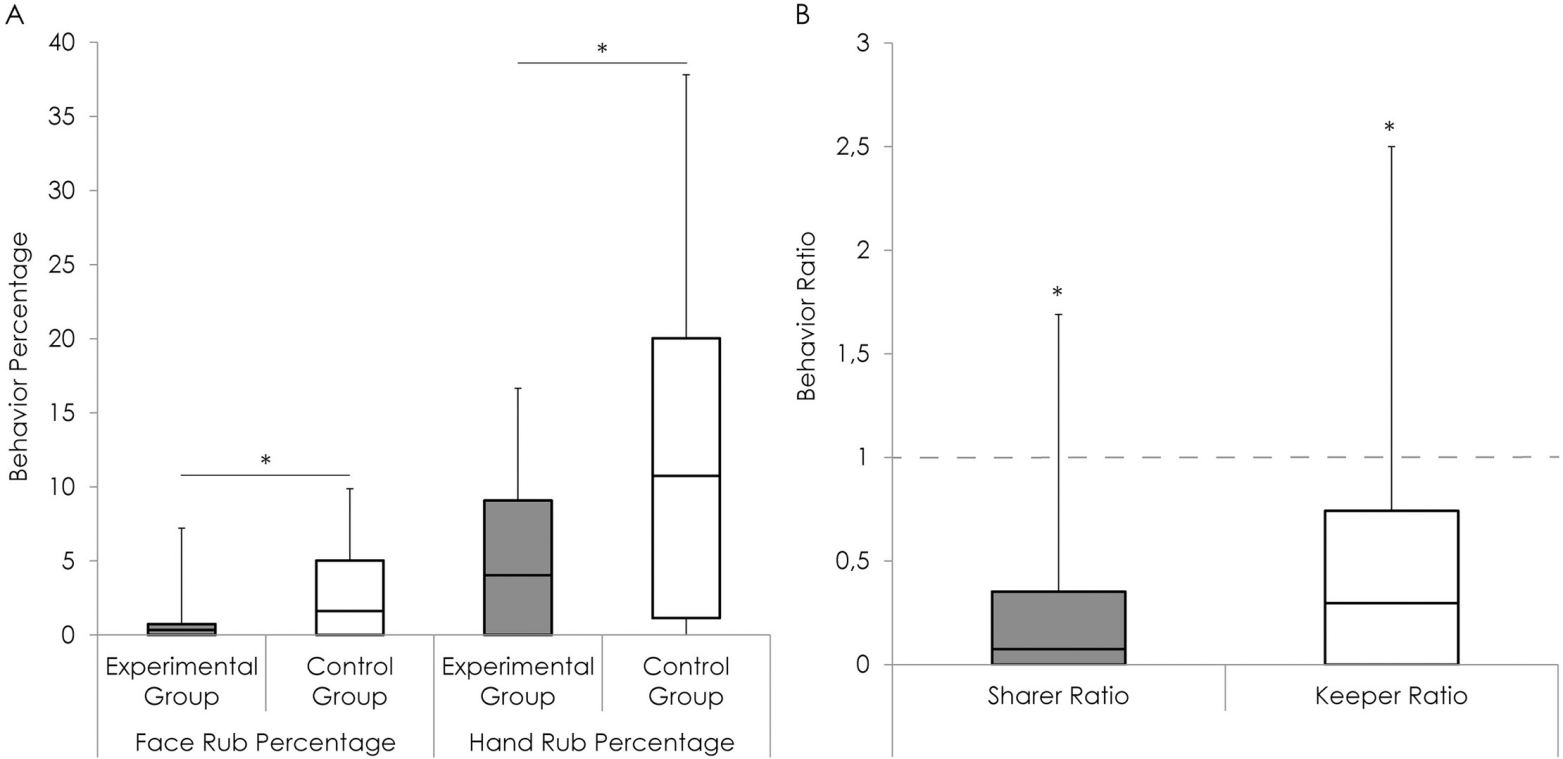
Mimicry and social mimicry box plots. A: Experimental and control groups’ face and hand rub percentages. Behavior percentages indicate the percentage of the story session that children performed the behavior. B: The experimental group’s sharer ratio and keeper ratio. Behavior ratios indicate the proportion of the experimental group’s behaviors relative to the control group’s mean behavior percentage.

To test the first hypothesis that the experimental group mimicked the storytellers, the experimental group’s face rubbing and hand rubbing percentages were compared with those of the control group. For both behaviors, the control group performed the behaviors for a significantly higher percentage of time than the experimental group and these effects were of a medium effect size (Fig 2A). A Mann-Whitney U test revealed that the experimental group (Mdn = 0.34%) rubbed their face significantly less than the control group (Mdn = 1.61%), *U* = 209.5, *z* = -2.20, *p* = .028, *r* = .31. Likewise, hand rubbing was significantly lower in the experimental group (Mdn = 4.04%) than in the control group (Mdn = 10.75%), *U* = 200, *z* = -2.33, *p* = .02, *r* = .33. This effect remained significant for both behaviors when the ten children of the experimental group who reported having noticed one or both of the behaviors were excluded; face rubbing: *U* = 119, *z* = -2.370, *p* = .018, *r* = .37, hand rubbing: *U* = 118, *z* = -2.36, *p* = .019, *r* = .37.

The second hypothesis was tested within the experimental group. Behavior ratios of the sharer’s behavior were compared with those of the keeper to investigate social (negative) mimicry (Fig 2B). A Wilcoxon signed rank test indicated that, contrary to expectation, there was no significant difference between sharer ratios (Mdn = 0.07) and keeper ratios (Mdn = 0.30), *z* = -1.43, *p* = .153, *r* = .27. Instead, sharer ratios (*z* = -4.35, *p* < .001, *r* = .82) and keeper ratios (*z* = -3.24, *p* = .001, *r* = .61) were significantly lower than 1, as tested with one-sample Wilcoxon signed rank tests. Thus, on a group level, both the sharer’s and the keeper’s behaviors were negatively mimicked.

**Fig 3:**
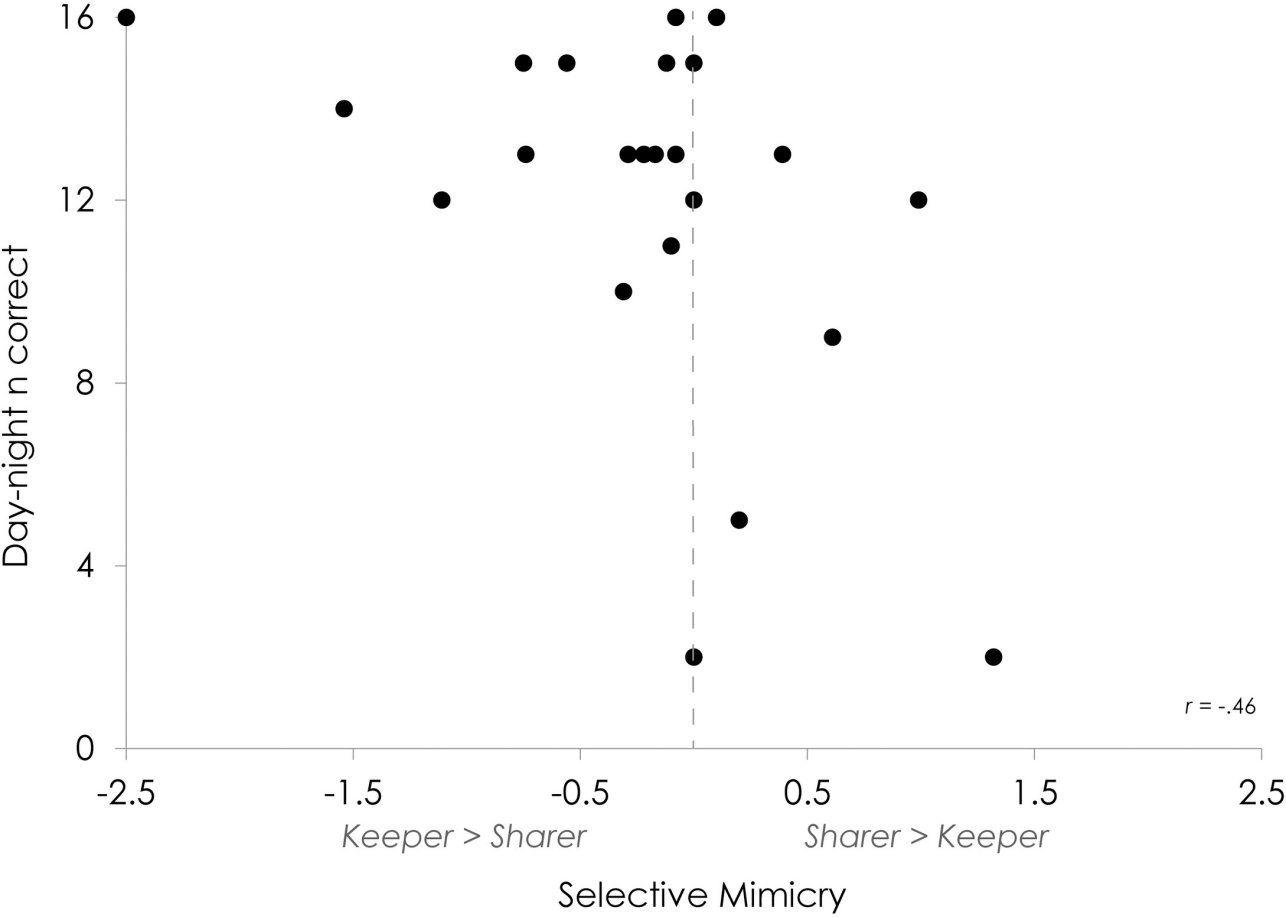
Relation between inhibitory control and selective mimicry.

The third and fourth hypotheses investigated individual differences. The third hypothesis concerned the effect of inhibitory control on selective mimicry (i.e. sharer minus keeper ratios) and the moderation of this effect by social understanding. This was investigated using a linear regression with a moderator. A linear regression model with the day-night score as the predictor yielded a model that significantly predicts selective mimicry (Table 1). Adding social understanding as a moderator of inhibitory control’s effect on selective mimicry did not significantly improve the model. The direction of the relation suggests that the better a child’s inhibitory control, the more negative the difference between sharer and keeper behavior ratios (i.e. the more they mimicked the keeper; Fig 3).

**Table 1:** Regression model inhibitory control and selective mimicry.

		B	SE B	β	R^2^
Step 1					
	Constant	0.884	0.457		
	Day-Night	-0.089	0.036	-.460*	0.211
Step 2					
	Constant	0.865	0.457		
	Day-Night	-0.088	0.036	-.460*	
Social Understanding Moderator		0.249	0.245	.188	0.247

*p = .021

Step 2: ΔR^2^ = 0.035, *p* = .32

The fourth hypothesis entailed the relation between social understanding and how much children mimicked overall. There was a significant positive correlation between children’s scores on the social understanding scale and the total mimicry ratios (i.e. the summed behavior percentages relative to the control group’s behavior percentages), *r*(26) = .32, *r*^*2*^ = .10, *p* = .048 (one-tailed). This indicates that the higher children’s social understanding, the more they performed the behaviors.

## Discussion

The aims of this study were to investigate 5-year-olds’ social behavioral mimicry in a naturalistic interaction and to explore the developmental factors contributing to this behavior. After a live social interaction in which a child interacted with a kind individual, an experimenter who shared a sticker, and an unkind individual, an experimenter who kept stickers for herself, children observed a video of these two experimenters each tell a short story. During the story session, the experimental group observed the experimenters rub their face and hands, while the control group did not see these behaviors. Following the stories, children completed the day-night task as a measure of inhibitory control. Parents’ evaluations of their child’s social understanding were collected through the CSUS.

There were four comparisons of interest: (1) behavior percentages in the experimental versus control groups to test for (negative) mimicry, (2) sharer versus keeper ratios to examine social mimicry effects, (3) the influence of inhibitory control on children’s selective mimicry of the sharer over the keeper, and (4) the relation between children’s social understanding and how much they (negatively) mimicked overall. Although group-level analyses showed surprising negative mimicry effects (hypotheses 1 and 2), investigations considering inhibitory control and social understanding did help shed light on the range of (negative) mimicry behaviors (hypotheses 3 and 4).

First, we investigated whether children displayed behavioral mimicry overall. The percentage of time children spent face rubbing and hand rubbing was compared between the two groups. Significant suppression was found for both behaviors; children in the experimental group, those who observed the experimenters perform behaviors, performed face and hand rubbing less than the control group. Importantly, this effect cannot be explained as a consequence of participants noticing the behaviors, as the findings held when the participants who reported noticing the behaviors during the debriefing were excluded from the analyses. Thus, overall, there was evidence for negative mimicry, with children seemingly refraining from these behaviors when they observed others carry them out.

The medium effect sizes of this suppression suggest that the findings are not spurious; instead, the sole difference between the experimental and control groups’ experience during the experiment, namely the presence or absence of the experimenters’ behaviors during the stories, seems to have caused a considerable difference in the two groups’ behavior. Yet, it is unclear why there would be an overall decrease in behaviors, since previous evidence for negative mimicry in adults was related to the social characteristics [11]. It could be the case that the presence of the to-be-mimicked behaviors subtly influenced the “interaction” dynamics in the experimental group to a degree of decreasing affiliation and thereby reducing affiliative mimicry. Live interactions are in constant flux, with both partners mutually adjusting to the other [29]. In this video-based design, though, the storytellers could not act contingently on the children’s behavior in any way. Resembling a context with too little mimicry [30], this lack of any contingency might have served as a disaffiliative signal towards the child [31], possibly reducing their mimicry behaviors as a result. Though plausible, this interpretation warrants further investigations, as previous mimicry studies have successfully utilized video-based methods before [9,11–13,23,32].

Conversely, the suppression effects might have been a consequence of a high behavior prevalence in the control group. Here, the storytellers did not perform any manual actions and, as was the case for the experimental group, they could also not act contingently on children’s behaviors through other means. Perhaps this relatively motionless storytelling contrasted too much with the dynamic live sticker game preceding the stories, making the children uncomfortable, causing them to fidget more, and, hence, thereby also perform the target behaviors more. However, past adult work in support of this design challenges this reason for the suppression effects. In a non-social 1-minute baseline preceding the experiment, behavior rates were three times higher than those of a matched baseline group like that used in the present study [23], thus speaking against an increased fidgeting account. Future research in which contingency between experimenter and child are better conserved between experimental and control groups will be necessary to tease apart these scenarios (i.e. decreased experimental group behaviors vs. increased control group behaviors) contributing to the negative mimicry effect.

The second comparison tested whether children’s (negative) mimicry was affected by the social manipulation. It was hypothesized that children’s behavior ratios for the sharer would be significantly higher than those for the keeper. In addition, it could be predicted that particularly sharer ratios would be higher than 1, as this would indicate more behavior in the experimental group than in the control group (i.e. mimicry), while keeper ratios might be lower than 1, indicating less behavior in the experimental than control group (i.e. negative mimicry). However, no difference was found between sharer and keeper ratios. Moreover, both sharer and keeper ratios were significantly lower than 1, indicating that, on a group level, children suppressed their behaviors for both experimenters.

A negative mimicry effect has been previously documented in an adult study in which the more participants disliked the confederates the more negative mimicry they displayed. Yet, in that study, liked confederates were mimicked [11]. In addition to the earlier discussed possible influences on total mimicry effects, the lack of group-level differences between the sharer and keeper in the present study could be due to the keeper’s inconsistent behavior. During the warm-up period, the keeper and sharer interacted to an equal and positive extent with the child, and later, following the sticker game, the keeper appeared to cooperate with the sharer by sitting next to her and telling the child a story. This might have caused children to reason about why the keeper kept both stickers leading them to overlook this unkind act and still want to affiliate. It could also have lead children to feel ostracized by this otherwise kind individual thereby possibly increasing affiliation goals (e.g. [17,33]). In these situations, children would hold affiliation goals with both the keeper and sharer, thus explaining why there was no difference in children’s negative mimicry of the two experimenters.

Whereas the explicit liking measure did not show a significant preference for the sharer, this finding in itself does not necessarily indicate that the social manipulation was not understood by the children. The phrasing of the preference measure, namely asking the child with whom they wanted to play if they could play another game, might have been interpreted differently in the context of this experiment than in previous experiments in which it was deemed understandable for children of this age [34]. At the end of the experiment, several children reasoned that they selected the keeper to try to get the sticker from her, indicating that the question might have been interpreted as a repetition of the sticker game. Thus, the liking measure was not a sensitive measure of children’s true preferences. Indeed, all children could correctly identify the keeper at the end of the experiment, so there is little reason for children not to have been sensitive to fairness, an effect demonstrated in several past studies [19–21,35]. However, the lack of an effect of the social manipulation on children’s (negative) mimicry, as well as the overall negative mimicry effects, remains up for debate. It could be the case that the use of behavioral mimicry in interactions, and particularly its sensitivity to social dynamics, is still developing during early and middle childhood. Hence, whereas some strong social effects already influence mimicry on a group level at the age of five, such as group boundaries [12], more subtle or intricate social dynamics might only be visible across children later during development.

Evidence in favor of a prolonged developmental trajectory of social mimicry comes from the third analysis, entailing the effect of inhibitory control on selective mimicry. Even though there were no statistically significant social effects on the group level, individual differences within the groups could still have affected children’s selective mimicry. Accordingly, we investigated whether inhibitory control influences selective mimicry and whether this effect is moderated by children’s social understanding. In a regression analysis, inhibitory control significantly predicted selective mimicry, but the addition of social understanding as a moderator did not improve the predictive power of the model. The model indicates that the more inhibitory control a child had, the more negative the difference between sharer and keeper ratios was. In other words, while children with lower inhibitory control would generally suppress the sharer’s behavior less than the keeper’s (i.e. mimic the sharer more), children with higher inhibitory control would overall suppress the keeper’s behavior less than the sharer’s (i.e. mimic the keeper more). This relation suggests that, if possible based on their inhibitory control, children influenced their behaviors in favor of the keeper. Though speculative, this reasoning is in line with the enhanced affiliation goals for the keeper discussed above. Irrespective of the precise social motivations, these results provide the first indication that mimicking during childhood might require some level of behavioral control.

Finally, there was a significant correlation between social understanding and total mimicry in the hypothesized direction; the higher children’s social understanding, the more behaviors they carried out. In other words, the children with higher social understanding scores were also generally the children who suppressed their behaviors less, and a few would even be said to have mimicked as their total mimicry ratios were above 1. This is in line with the idea that a general understanding of social interactions and the individuals in those interactions is important for the display of affiliative behaviors. That is, it could be that through increasing social experience and understanding, children’s mimicry becomes increasingly multifaceted. However, the amount of explained variance (approximately 10%) is arguably quite low, indicating that this finding, though in line with the a priori hypothesis, should be interpreted cautiously. The range of the CSUS scores might have limited the strength of the effects of social understanding. The CSUS scale ranges from 1 to 4 but the lowest individual score in the present sample was 2.5, meaning that no participant scored in the lower half of the scale. This lack of spread might also have limited the statistical sensitivity of finding the hypothesized moderator role of CSUS on inhibitory control’s influence on selective mimicry. Future research should investigate individual differences and their effects on children’s development of mimicry in more diverse samples and different age groups to better understand the relations documented in this study.

In conclusion, this study was designed to measure social mimicry in young children and investigate the effects of developmental factors on mimicry’s emergence. Only suppression, or negative mimicry, effects were found, regardless of the social identity of the interaction partner. On an individual level, inhibitory control predicted the amount of suppression of a sharer’s versus keeper’s behaviors. Additionally, social understanding was related to how much children mimicked overall. Thus, though the findings were limited, this study provides the first evidence that the occurrence of social behavioral mimicry during childhood is likely a product of both social and cognitive factors. Moreover, the novel design presented here provides a naturalistic yet controlled means through which to investigate behavioral mimicry from toddlerhood to middle childhood. Taken together, this study opens the door for further investigations into the development of mimicry in early and also later childhood, the social contexts in which negative mimicry occurs, and the roles of behavior regulation and social understanding in the development of social interactive behaviors.

## Supporting information

S1 Table Behavior measure calculations. (PDF)

S1 File Data file with reported measures. (XLSX)

S2 File Segment of a stimulus video. (AVI)
