# Supplementary material for: Modulating mimicry: Exploring the roles of inhibitory control and social understanding in 5-year-olds' behavioral mimicry
Source: PLoS One. 2018 Mar 7;13(3):e0194102. doi: 10.1371/journal.pone.0194102 (PMC5841819; doi:10.1371/journal.pone.0194102)
Supplement: S1 Table — (PDF) [file pone.0194102.s001.pdf]

S1 Table. Behavior Measure Calculations

Measure	Group	Calculation	Comparison
Behavior Percentages<br>Face rub percent<br>Hand rub percent	experimental, control	time spent carrying out behavior / time watching story videos	Hypothesis 1: experimental vs. control group
Behavior Ratios<br>*Face rub ratio*<br>*Hand rub ratio*	*experimental*	*behavior % / control group's mean behavior%*	*Intermediate measure*
Social Ratios<br>Sharer ratio<br>Keeper ratio	experimental	behavior ratios split into sharer's and keeper's behaviors (dependent on counterbalancing)	Hypothesis 2: sharer vs. keeper ratios
Selective Mimicry	experimental	sharer ratio - keeper ratio	Hypothesis 3: relation inhibitory control and selective mimicry
Total Mimicry Ratio	experimental	Σ behavior %s / control group's mean Σ behaviors %s	Hypothesis 4: correlation social understanding and total mimicry
